# Supplementary material for: The interactome of intact mitochondria by cross-linking mass spectrometry provides evidence for coexisting respiratory supercomplexes
Source: Mol Cell Proteomics. 2017 Dec 8;17(2):216–32. doi: 10.1074/mcp.RA117.000470 (PMC5795388; doi:10.1074/mcp.RA117.000470)
Supplement: Supplemental Data [file supp_RA117.000470_133922_0_supp_23978_fzffwf.zip › spectra_annotation/mito_spectra_annotation/180-1-15-1-5-1.pdf]

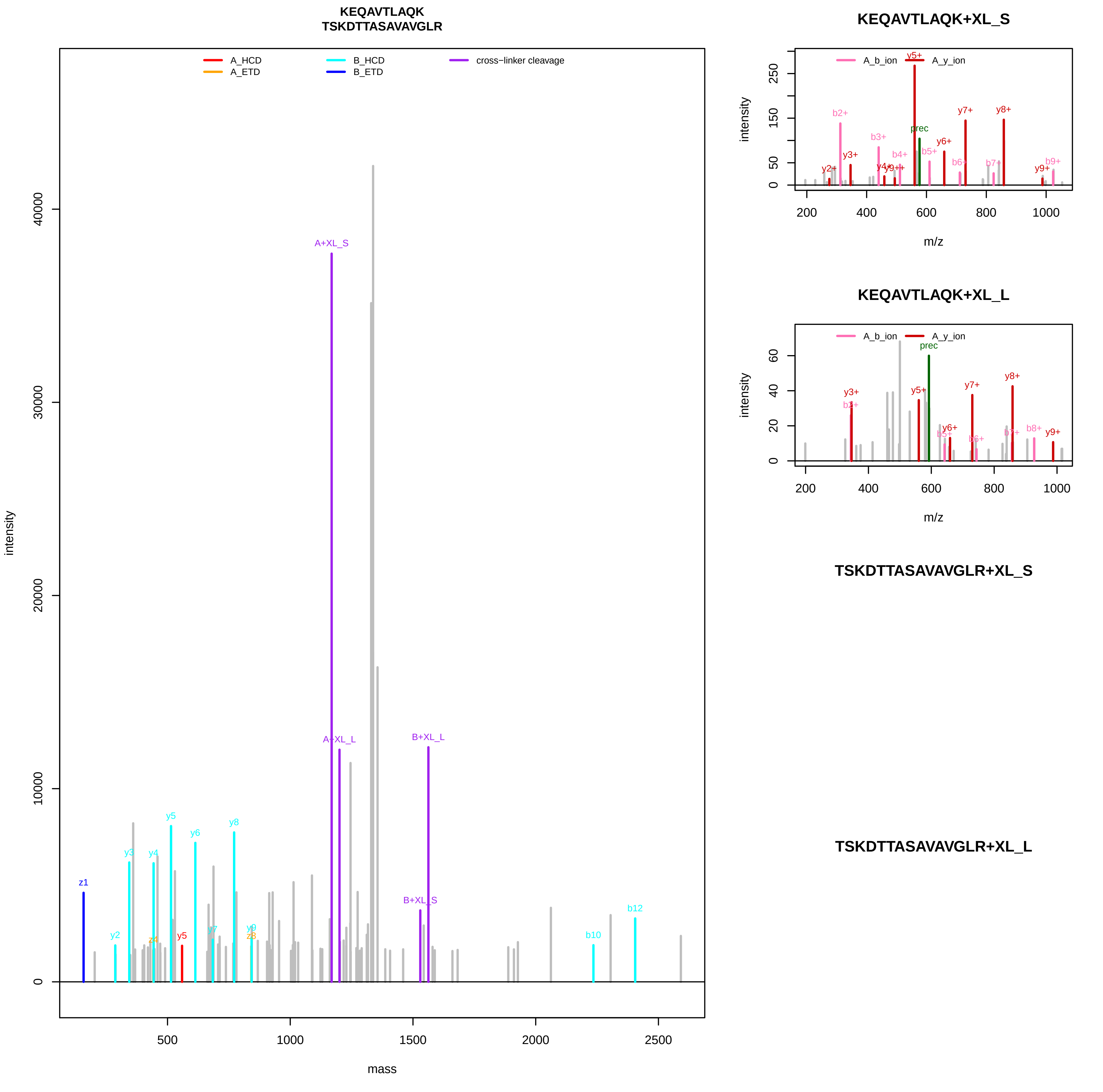
KEQAVTLAQK
TSKDTTASAVAVGLR
A_HCD
A_ETD
B_HCD
B_ETD
cross−linker cleavage
A+XL_S
A+XL_L
B+XL_L
B+XL_S
z1
y2
y3
y4
z4
y5
y6
y7
y8
y9
z8
b10
b12
intensity
mass
0
10000
20000
30000
40000
500
1000
1500
2000
2500
KEQAVTLAQK+XL_S
A_b_ion
A_y_ion
b2+
y2+
y3+
b3+
y4+
y9++
b4+
y5+
prec
b5+
y6+
b6+
y7+
b7+
y8+
y9+
b9+
intensity
m/z
0
50
150
250
200
400
600
800
1000
KEQAVTLAQK+XL_L
A_b_ion
A_y_ion
prec
y3+
b2+
y5+
y6+
b5+
y7+
b6+
y8+
b7+
b8+
y9+
intensity
m/z
0
20
40
60
200
400
600
800
1000
TSKDTTASAVAVGLR+XL_S
TSKDTTASAVAVGLR+XL_L
